# Supplementary material for: Immunogenicity of a Candidate DTacP-sIPV Combined Vaccine and Its Protection Efficacy against Pertussis in a Rhesus Macaque Model
Source: Vaccines (Basel). 2021 Dec 30;10(1):47. doi: 10.3390/vaccines10010047 (PMC8779802; doi:10.3390/vaccines10010047)
Supplement: Supplementary file 1 [file vaccines-10-00047-s001.zip › Supplementary Figure S1.pdf]

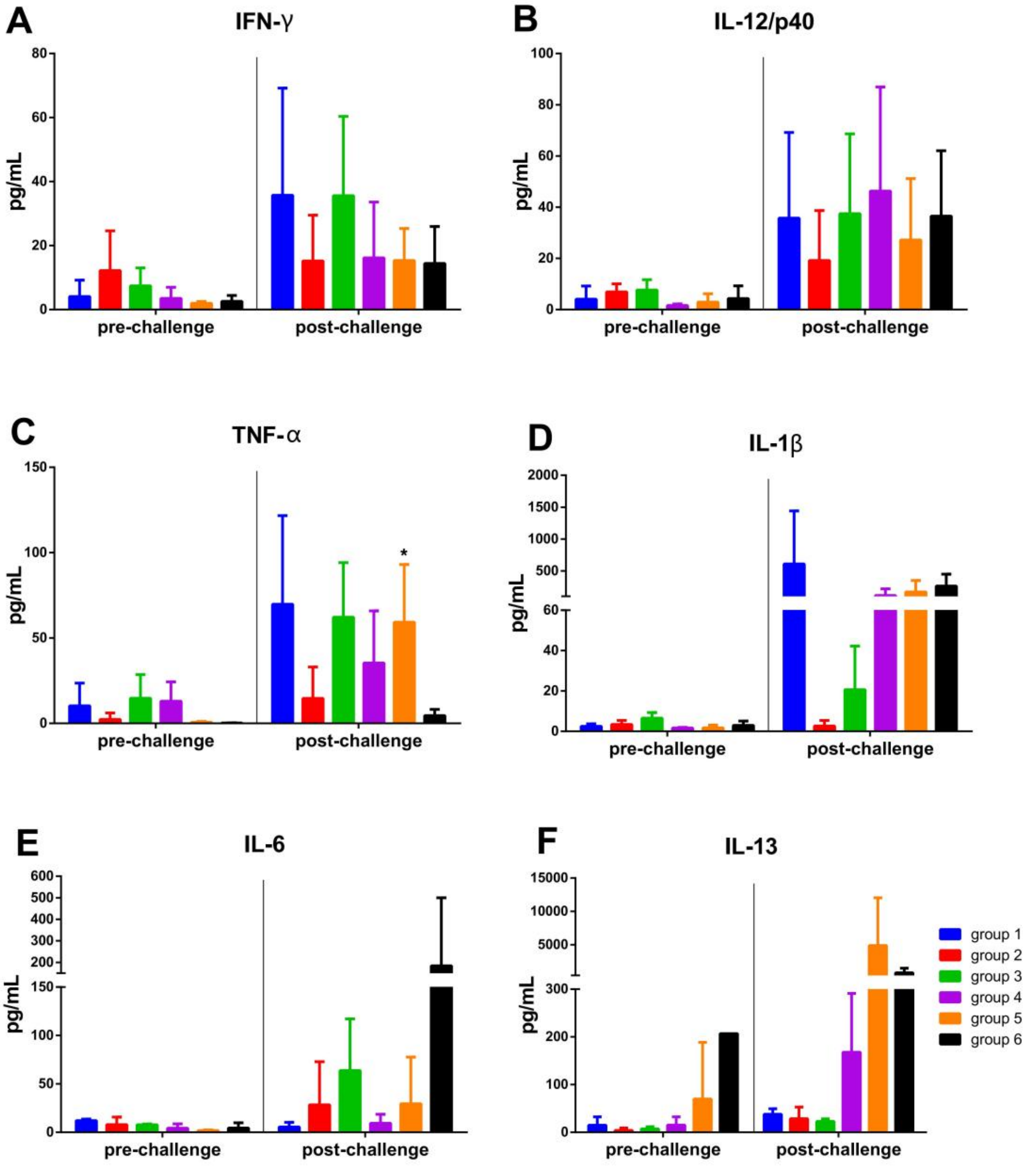

Figure. S1 Dynamic profiles of cytokines after challenge. The differences between the pre-challenge and peak cytokine production post-challenge period (3, 7, 14, 21, 28 days after challenge) were compared. Cytokines, including (A) IFN-γ, (B) IL-12/P40, (C) TNF-α, (D) IL-1β, (E) IL-6, and (F) IL-13, were tested. The results are shown as the mean with SEM. Data were analyzed using an unpaired *t*-test. * $P < 0.05$ (n = 3).
